# Relationships between the acoustic startle response and prepulse inhibition in C57BL/6J mice: a large-scale meta-analytic study

**DOI:** 10.1186/s13041-018-0382-7

**Published:** 2018-07-13

**Authors:** Hirotaka Shoji, Tsuyoshi Miyakawa

**Affiliations:** Division of Systems Medical Science, Institute for Comprehensive Medical Science, Fujita Health University, 1-98 Dengakugakubo Kutsukake-cho, Toyoake, Aichi 470-1192 Japan

**Keywords:** Acoustic startle response, Prepulse inhibition, Meta-analysis, Behavioral test battery, Mouse phenotype database

## Abstract

Prepulse inhibition (PPI) is the suppression of a startle reflex response to a startle stimulus that occurs when a weak prepulse stimulus precedes the startle stimulus. PPI is measured to assess sensorimotor gating across species, including humans and rodents. Reduced PPI, which is thought to reflect dysfunction of sensorimotor gating, is reported in patients with psychiatric disorders, such as schizophrenia, bipolar disorder, and post-traumatic stress disorder (PTSD), and in animal models of these disorders. Individual differences in basal startle reactivity occur even in a genetically homogenous group of animals; however, there is limited information regarding whether basal levels of the startle response are associated with variations in PPI levels. Here, to explore the relationship between an acoustic startle response (ASR) and PPI, we performed a meta-analysis of data obtained from more than 1300 C57BL/6J male mice on the influence of an ASR to 110- and 120-dB startle stimuli on the PPI levels of the ASR at 74- and 78-dB prepulse intensities. Examination of scatter plots of the ASR amplitudes and PPI levels followed by correlation analyses indicated that there is no simple linear relationship between the two measures; when mice were divided into three groups on the basis of their startle amplitudes, there were positive correlations between the amplitude of the ASR to the 110-dB stimulus and PPI levels in a group of mice that showed lower ASR amplitudes among the genetically homogenous group, whereas no significant correlations were identified in groups of mice that showed intermediate and higher ASR amplitudes. As indicated by the correlation analysis, the lowest responders to the 110-dB stimulus exhibited lower levels of PPI than the intermediate or higher responders. In contrast, for the 120-dB stimulus, a negative correlation was identified between the amplitude of the ASR to the 120-dB stimulus and the PPI levels in the groups of mice that showed intermediate or higher ASR amplitudes. Lower and intermediate responders showed higher levels of PPI than higher responders to the 120-dB stimulus. These findings suggest that basal startle reactivity may affect PPI levels in male C57BL/6J mice, thus representing one potential confounding factor that may confuse the interpretation of PPI results. These findings emphasize the importance of careful examination of startle reactivity to ensure a reliable assessment of PPI.

**Electronic supplementary material:**

The online version of this article (10.1186/s13041-018-0382-7) contains supplementary material, which is available to authorized users.

## Introduction

Prepulse inhibition (PPI) is a phenomenon in which a weak sensory stimulus suppresses a startle response caused by a sudden intense stimulus [1]. PPI is thought to reflect sensorimotor gating, a form of central nervous system inhibition in which irrelevant sensory information is filtered out during the early stages of processing so that attention may be focused on more salient features of the environment [2, 3]. Reduced PPI of the startle response has been extensively demonstrated in patients with schizophrenia [1, 2, 4–6] and potentially in patients with other psychiatric and neurological disorders, such as obsessive compulsive disorder (OCD) [7], Huntington’s disease [8], Tourette syndrome [9], panic disorder [10], and post-traumatic stress disorder (PTSD) [11], which suggests sensorimotor gating deficits in these patients.

A PPI test is a widely used paradigm that enables the assessment of sensorimotor gating across species, including humans and rodents, in a similar fashion [12]. In rats and mice, the startle reflex response is measured as a whole-body flinch that may be elicited by an acoustic or tactile (air puff) startle-eliciting stimulus similar to stimuli used in humans. The PPI of the startle response occurs when a weak prepulse stimulus is presented 30–500 ms prior to the startling stimulus. In general, 100- to 120-dB sound stimuli are used as the startling stimulus to measure the acoustic startle response (ASR), and an acoustic prepulse is presented at an intensity of 4–16 dB above a continuous background noise, e.g., 70-dB white noise, to evaluate PPI of the ASR. For the analysis of each animal, the percent PPI is calculated as the percent decrease in the startle amplitude at each prepulse intensity relative to the startle-alone trial. Animal studies have indicated that genetic and pharmacological manipulations may alter the ASR and PPI, which demonstrates that the PPI test is a useful tool for investigating the underlying mechanisms of sensory inhibitory processing in the brain, as well as translational research of neuropsychiatric disorders [6, 13].

The ASR and PPI may be affected by genetic, biological, and environmental factors, such as strain [14, 15], age [16, 17], sex [18, 19], and housing conditions [20, 21]. Various experimental factors and other unidentified factors potentially lead to individual or group differences in the startle response in animals. In these cases, the interpretation of PPI differences between groups may be complicated by the differences in the basal level of the startle response. Therefore, researchers have investigated whether baseline ASR magnitudes affect PPI levels. Ison et al. [22] reported that although the percent PPI of the startle reflex did not differ between CBA/J mice with high-startle baselines and low-startle baselines, there was a weak positive correlation between the basal startle response and percent PPI. Similar results were obtained in C57BL/6J mice that exhibited low or intermediate startle amplitudes in a group under testing conditions, whereas the startle reactivity was not correlated with the percent PPI in mice with high startle amplitudes [23]. Although the previous studies, based on a small number of animals, suggest that the association of basal startle reactivity with behavioral responses reflects an inhibitory mechanism of sensory information processing, a paucity of information remains regarding the relationship between ASR and PPI.

In our laboratory, we have analyzed various types of behavior in more than 180 strains of genetically engineered mice in a battery of behavioral tests with our standardized procedure [17, 24, 25]. In the PPI test, we used several different combinations of a startle stimulus and a prepulse stimulus to identify potential phenotypes in ASR and PPI. The mice were exposed to two different intensities of startle stimulus (i.e., 110- or 120-dB stimulus) with 74-dB, 78-dB, or no prepulse stimulus in a test session. In this study, we used the behavioral data of more than 1300 wild-type C57BL/6J male mice subjected to the PPI test with data stored in our database and examined the relationships between ASR amplitudes and PPI levels using this substantial amount of data from a genetically homogeneous group of mice.

## Methods

### Animals and experimental design

Genetically engineered mice and their wild-type control mice were transported from the animal facilities of other laboratories or vendors to the facilities of our laboratory (for detailed information on individual mice included in the studies previously published [17], refer to the Mouse Phenotype Database, URL: http://www.mouse-phenotype.org). The mice were subjected to a battery of behavioral tests with our standardized protocol (for details, refer to Shoji et al. [17]). We used behavioral data of the startle response/PPI test that were obtained from 1363 wild-type control male mice 2–12 months old (2–3 months old, *n* = 757; 4–5 months old, *n* = 389; 6–7 months old, *n* = 167; 8–12 months old, *n* = 50). The wild-type mice were derived from a C57BL/6J strain and its substrains (6JJcl or 6JJmsSlc) maintained in Japan, and more than 90% of the mice were backcrossed at least six times (and more than 95% of the mice used were backcrossed at least five times) with C57BL/6J mice. Thus, the genetic backgrounds of the mice were regarded as “C57BL/6J”. They were housed in plastic cages with paper bedding (Paper Clean; Japan SLC, Inc., Shizuoka, Japan) under a 12-h light/dark cycle (lights on at 7:00 a.m.) with access to food (CRF-1; Oriental Yeast Co., Ltd.) and water *ad libitum*. Behavioral testing was performed between 9:00 a.m. and 6:00 p.m. The testing apparatuses were cleaned with super hypochlorous water and 70% ethanol to remove olfactory stimuli after each test. All behavioral testing procedures were approved by the Animal Care and Use Committee of Kyoto University Graduate School of Medicine and the National Institute for Physiological Sciences in Japan.

### Startle response/PPI test

A startle reflex measurement system (O’Hara & Co., Tokyo, Japan) was used to measure the ASR to a loud noise and PPI of the startle response, as previously described [17]. A 20-min test session was initiated by placing a mouse in a plastic cylinder in a sound-attenuating chamber, in which the mouse was left undisturbed for the first 10-min period and was subsequently subjected to startle-stimulus-only trials and PPI trials for 10 min. The last 10-min session consisted of six trial types, i.e., two types of startle-stimulus-only trials (110 or 120 dB auditory stimulus) and four types of PPI trials (74 dB prepulse + 110 dB stimulus, 74–110 dB; 78 dB prepulse + 110 dB stimulus, 78–110 dB; 74 dB prepulse + 120 dB stimulus, 74–120 dB; and 78 dB prepulse + 120 dB stimulus, 78–120 dB). White noise (40 ms) was used as the startle stimulus for all trial types. A background white noise was presented at a level of 70 dB during the test. The prepulse sound was presented for 20 ms at an intensity of 74 or 78 dB 100 ms prior to the presentation of the startle stimulus. The startle response was recorded for 400 ms starting with the onset of the startle stimulus using the startle reflex measurement system with an accelerometer mounted below the cylinder. Six blocks of the six trial types were presented to the mice in a pseudo-random order, and each trial type was presented once within a block. The average inter-trial interval was 15 s with a range of 10–20 s. The peak startle amplitude was recorded for use as a dependent variable. The percent PPI was calculated for analysis for each mouse according to the following formula: the percentage of PPI = 100 × [1 - (startle response for prepulse + startle trial)/(startle response for startle stimulus alone trial)].

### Data analysis

Statistical analysis was conducted with statistical software (SAS University Edition; SAS Institute, Cary, NC, USA). Behavioral data were analyzed using a Spearman rank correlation test and Kruskal-Wallis test followed by post hoc comparisons with the Dwass-Steel-Critchlow-Fligner test to avoid potential statistical problems with non-normal distributions. The statistical significance level was set at *p* < 0.05.

## Results

**Fig. 1:**
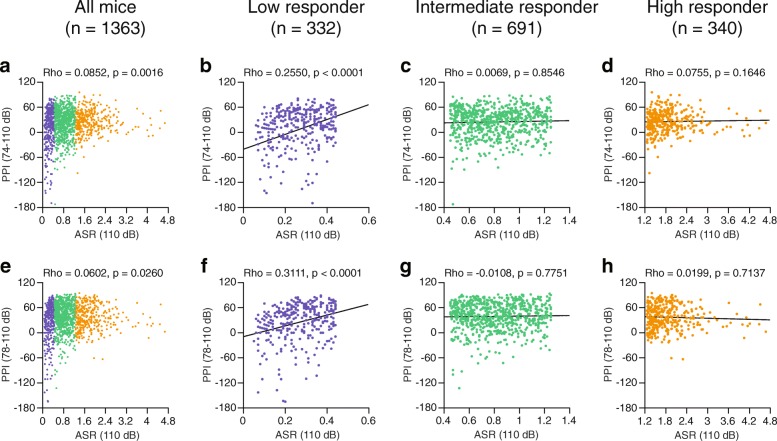
Scatter plots of the amplitudes of the startle response to a 110-dB pulse stimulus and percentages of prepulse inhibition of the acoustic startle response in male C57BL/6J mice. Relationships between acoustic startle responses to 110-dB pulse stimulus and percentages of prepulse inhibition were assessed by Spearman’s rank correlation coefficients (Rho) and *p* values at 74 dB prepulse + 110 dB stimulus trial (74–110 dB) and 78 dB prepulse + 110 dB stimulus trial (78–110 dB) in 1363 mice (**a**, **e**­). The mice were divided into low (**b**, **f**), intermediate (**c**, **g**), and high (**d**, **h**) startle responder groups on the basis of their startle amplitudes. In each responder group, relationships between the acoustic startle response and percentages of prepulse inhibition were assessed by calculating the correlation coefficients (Rho) and p values for 74–110 dB trials (**b**–**d**) and 78–110 dB trials (**f**–**h**)

**Fig. 2:**
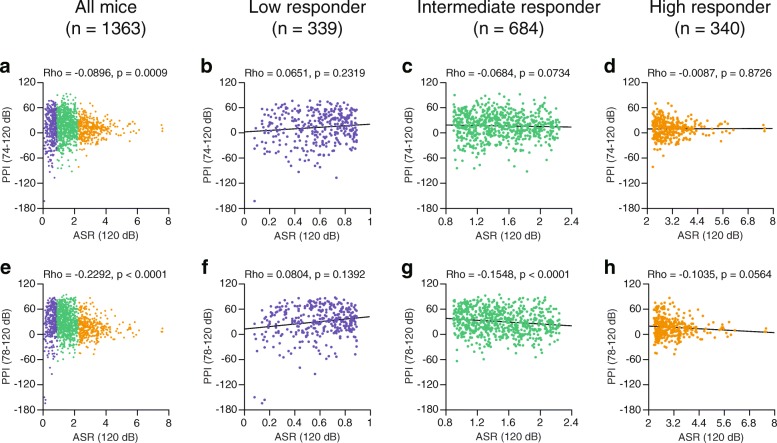
Scatter plots of the amplitudes of the startle responses to a 120-dB pulse stimulus and percentages of prepulse inhibition of the acoustic startle response in male C57BL/6J mice. Relationships between acoustic startle response to 120-dB pulse stimulus and percentages of prepulse inhibition were assessed by Spearman’s rank correlation coefficients (Rho) and p values for a 74 dB prepulse + 120 dB stimulus trial (74–120 dB) and 78 dB prepulse + 120 dB stimulus trial (78–120 dB) in 1363 mice (**a**, **e**­). The mice were divided into low (**b**, **f**), intermediate (**c**, **g**), and high (**d**, **h**) startle responder groups on the basis of their startle amplitudes. In each responder group, relationships between the acoustic startle response and percentages of prepulse inhibition were assessed by calculating the correlation coefficients (Rho) and p values for the 74–120 dB trial (**b–d**) and 78–120 dB trial (**f**–**h**)

To examine the relationships between the basal ASR and PPI of the ASR, we initially conducted correlation analyses with Spearman’s method between the amplitudes of the ASR to the 110- or 120-dB stimulus and the percentages of PPI of the ASR in the group of 1363 C57BL/6J mice (Figs. 1 and 2, left panels). There were small and significant positive correlations between the amplitude of the ASR to the 110-dB stimulus and the percent PPI when a 74- or 78-dB prepulse was presented to the group (Fig. 1a, for 74–110 dB, Rho = 0.0852, *p* = 0.0016; Fig. 1e, for 78–110 dB, Rho = 0.0602, *p* = 0.0260). In contrast, significant small negative correlations were identified between the amplitude of the ASR to the 120-dB stimulus and the percent PPI at the presentations of 74- and 78-dB prepulses (Fig. 2a, for 74–120 dB, Rho = − 0.0896, *p* = 0.0009; Fig. 2e, for 78–120 dB, Rho = − 0.2292, *p* < 0.0001).

In the genetically homogeneous group of mice, as illustrated in Figs. 1 and 2, the scatter plots of the ASR amplitudes and PPI levels showed that there were no simple linear relationships between the two measures. Differences in the ASR amplitudes among the mice were identified in response to each startle stimulus; the ASR amplitudes ranged from 0.05 to 4.67 at the 110-dB pulse stimulus (first quartile [Q1] = 0.45, median [second quartile, Q2] = 0.8, and third quartile [Q3] = 1.25) and 0.08 to 7.59 at the 120-pulse stimulus (Q1 = 0.9, Q2 = 1.44, and Q3 = 2.24). To further investigate the detailed relationships between the ASR and PPI in groups with different ranges of ASR amplitudes, we divided the 1363 mice into three groups on the basis of their ASR amplitudes at each startle stimulus (see, Table 1 and Additional file 1): the low-responder group (mice that exhibited startle amplitudes of less than the Q1), the intermediate-responder group (mice that exhibited startle amplitudes that ranged from the Q1 value to the Q3 value), and the high-responder group (mice that exhibited startle amplitudes of more than the Q3 value). When correlation analyses were performed in each subgroup, significant positive correlations were identified in the low-responder group between the amplitude of the ASR to the 110-dB stimulus and the percent PPI for both the 74–110 dB trial (Fig. 1b, Rho = 0.2550, *p* < 0.0001) and the 78–110 dB trial (Fig. 1f, Rho = 0.3111, p < 0.0001); there were no significant correlations for each trial in the intermediate- and high-responder groups (Fig. 1c, d, g, and h). At the intensity of 120-dB, there were no significant correlations between the ASR amplitudes in response to the 120-dB stimulus and the percent PPI for the 74–120 dB and 78–120 dB trials in the low-responder group (Fig. 2b, for 74–120 dB, Rho = 0.0651, *p* = 0.2319; Fig. 2f, for 78–120 dB, Rho = 0.0804, *p* = 0.1392). In the intermediate-responder group, there was a small negative correlation between the amplitude of the ASR to the 120-dB stimulus and the percent PPI at 74–120 dB; however, the result did not reach statistical significance (Fig. 2c, Rho = − 0.0684, *p* = 0.0734), and a significant negative correlation was identified between the amplitude of the ASR to the 120-dB stimulus and the percent PPI at 78–120 dB (Fig. 2g, Rho = − 0.1548, *p* < 0.0001). In the high-responder group, there was no correlation at 74–120 dB (Fig. 2d, Rho = − 0.0087, *p* = 0.8726); however, there was a trend towards a negative correlation at 78–120 dB (Fig. 2h, Rho = − 0.1035, *p* = 0.0564). When the intermediate- and high-responder groups, which showed a similar pattern of correlations between the ASR amplitudes and PPI levels, were combined, significant negative correlations were identified for each trial (for 74–120 dB, Rho = − 0.1551, *p* < 0.0001; for 78–120 dB, Rho = − 0.2845, *p* < 0.0001).

**Table 1:** Groups of mice used for correlation analyses between the ASR amplitudes and percent PPI

Group	110-dB startle-stimulus only trial		120-dB startle-stimulus only trial	
	Subgroup	Startle amplitude	Subgroup	Startle amplitude
Low responder	Group 1	ASR < 0.26 (12.5 percentile)	Group 1′	ASR < 0.61 (12.5 percentile)
	Group 2	0.26 (12.5 percentile) ≤ ASR < 0.45 (25 percentile)	Group 2′	0.61 (12.5 percentile) ≤ ASR < 0.9 (25 percentile)
Intermediate responder	Group 3	0.45 (25 percentile) ≤ ASR < 0.62 (37.5 percentile)	Group 3’	0.9 (25 percentile) ≤ ASR < 1.16 (37.5 percentile)
	Group 4	0.62 (37.5 percentile) ≤ ASR < 0.8 (50 percentile)	Group 4’	1.16 (37.5 percentile) ≤ ASR < 1.44 (50 percentile)
	Group 5	0.8 (50 percentile) ≤ ASR < 0.99 (62.5 percentile)	Group 5’	1.44 (50 percentile) ≤ ASR < 1.76 (62.5 percentile)
	Group 6	0.99 (62.5 percentile) ≤ ASR ≤ 1.25 (75 percentile)	Group 6’	1.76 (62.5 percentile) ≤ ASR ≤ 2.24 (75 percentile)
High responder	Group 7	1.25 (75 percentile) < ASR ≤ 1.725 (87.5 percentile)	Group 7’	2.24 (75 percentile) < ASR ≤ 2.93 (87.5 percentile)
	Group 8	1.725 (87.5 percentile) < ASR	Group 8’	2.93 (87.5 percentile) < ASR

**Fig. 3:**
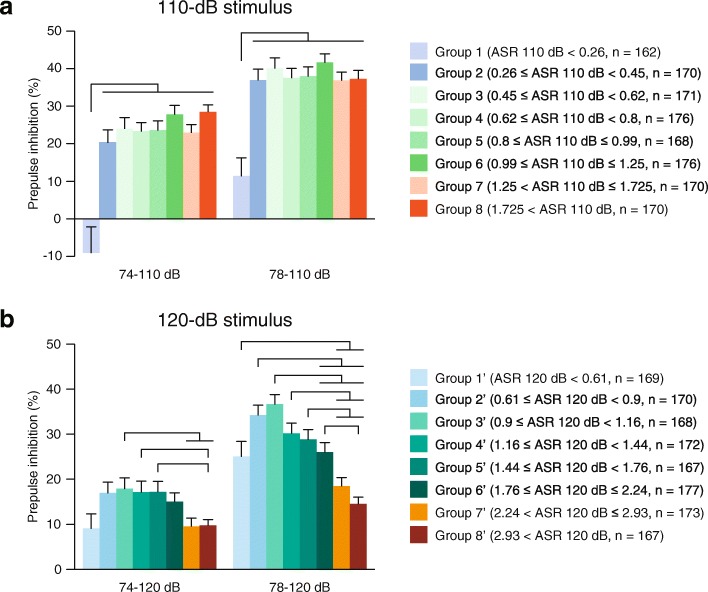
Comparisons of percentages of prepulse inhibition of startle responses between mice with different startle amplitudes. Mice were divided into eight groups based on the amplitudes of their startle responses to 110-dB and 120-dB stimuli at each 12.5 percentile of the distribution (for the 110-dB stimulus, Groups 1–8; for the 120-dB stimulus, Groups 1′–8′). Percentages of prepulse inhibition of startle responses are presented for each group of mice showing different startle amplitudes in response to the 110-dB stimulus (**a**) and 120-dB stimulus (**b**). The statistical results for comparisons among the groups are shown in Additional file 1

To clarify the differences in the PPI among the groups, the mice were further divided into eight subgroups based on their ASR amplitudes in response to 110-dB and 120-dB startle stimuli at each 12.5 percentile of the distribution, as described in Table 1: the lower and upper halves of the low-responder group (for the 110-dB stimulus, Groups 1 and 2; for the 120-dB stimulus, Groups 1′ and 2′), the four subgroups of the intermediate-responder group (for the 110-dB stimulus, Groups 3, 4, 5, and 6; for the 120-dB stimulus, Groups 3′, 4′, 5′, and 6′), and the lower and upper halves of the high-responder group (for the 110-dB stimulus, Groups 7 and 8; for the 120-dB stimulus, Groups 7′ and 8′). Comparisons of the PPI among the subgroups of responders to the 110-dB stimulus showed that there was a significant effect of group on PPI at 74–110 dB (χ^2^ = 36.3017, *p* < 0.0001) and 78–110 dB (χ^2^ = 46.1949, *p* < 0.0001) (Fig. 3a). Post hoc comparisons between the groups indicated that in the 74–110 dB and 78–110 dB trials, the mice in Group 1 exhibited a significantly lower PPI than the mice in the other groups (Group 1 < Groups 2, 3, 4, 5, 6,7, and 8, all *p* < 0.01, Dwass-Steel-Critchlow-Fligner test), and there were no significant differences in PPI among all groups with the exception of Group 1 (for the detailed statistical results of the Kruskal-Wallis test and Dwass-Steel-Critchlow-Fligner test, refer to Additional file 1).

Significant differences in PPI among the eight subgroups of responders to the 120-dB stimulus were identified for each trial type (for 74–120 dB, χ^2^ = 26.3291, *p* = 0.0004; for 78–120 dB, χ^2^ = 97.5232, p < 0.0001) (Fig. 3b and Additional file 1). The post hoc analyses indicated that Groups 7′ and 8′, the high startle responder groups, exhibited significantly lower percentages of PPI than the intermediate responder mice in Groups 3′, 4′ or 5′ in the 74–120 dB trial (Group 8′ < Groups 3′, 4′, and 5′; Group 7′ < Group 3′, all *p* < 0.05). In addition, in the 78–120 dB trial, the percentages of PPI in Groups ‘7 and 8’ were significantly lower than the percentages in Groups 1′, 2′, 3′, 4′, 5′, or 6′ (Groups 8′ < Groups 1′, 2′, 3′, 4′, 5′, and 6′; Group 7′ < Groups 1′, 2′, 3′, 4′, and 5′, all *p* < 0.05), and the percentage of PPI in Group 6′ was lower than the percentages in Groups 2′ and 3′ (Group 6′ < Groups 2′ and 3′, all *p* < 0.05). In general, mice that showed a low startle response to the 110-dB stimulus exhibited the lowest levels of PPI, and mice that showed a high startle response to the 120-dB startle stimulus displayed lower levels of PPI than mice with low or intermediate ASR amplitudes.

Latency to peak startle response is a relevant variable affecting ASR amplitudes and PPI levels. To further examine the relationships between ASR latency and ASR/PPI amplitudes, we performed correlation analyses with Spearman’s method between the latencies to the peak ASR with 110- or 120-dB stimulus and the percentages of PPI using data of 721 C57BL/6J mice that are available from the Mouse Phenotype Database (Additional file 2: Figure S1). The ASR latency to 110-dB stimulus was negatively correlated with ASR amplitude at 110-dB stimulus (Additional file 2: Figure S1A; Rho = − 0.2287, *p* < 0.0001) and PPI levels (Additional file 2: Figure S1B,C; for 74–110 dB, Rho = − 0.0742, *p* = 0.0463; for 78–110 dB, Rho = − 0.0885, *p* = 0.0175). Similarly, the ASR latency to 120-dB stimulus showed slight but significant negative correlation with PPI levels at 74–120 dB (Additional file 2: Figure S1E; Rho = − 0.0940, *p* = 0.0115), although the latency was not significantly correlated with ASR amplitudes at 120-dB stimulus (Additional file 2: Figure S1D; Rho = − 0.0220, *p* = 0.5537) nor PPI levels at 78–120 dB (Additional file 2: Figure S1F; Rho = − 0.0196, *p* = 0.5983). The inspection of the scatter plots in the ASR latency and PPI levels and correlation analyses indicate that mice showing a longer latency to respond startle stimuli tend to exhibit lower ASR amplitudes and PPI levels in most conditions that were used in our experiments.

## Discussion

This study aimed to investigate the relationships between an ASR to loud noise and the PPI of the startle response in adult C57BL/6J male mice. We performed a large-scale meta-analysis of the association of ASR amplitudes with percentages of PPI at different startle stimuli, with a focus on individual differences in the ASR amplitude in a genetically homogeneous group of 1363 C57BL/6J mice. Our study, which included a correlation analysis of ASR amplitudes and PPI levels, demonstrated that there was no simple linear relationship between ASR amplitudes and PPI levels, as indicated by weak positive or negative correlations between the two measures in the subgroups of mice with different ASR amplitudes in response to 110-dB and 120-dB stimuli. Moreover, positive correlations between the amplitude of the ASR to the 110-dB stimulus and PPI levels were identified in the low-responder group, whereas no significant correlations were identified in the intermediate- and high-responder groups at the 110-dB stimulus. At the higher intensity acoustic stimulus or 120-dB stimulus, there were no significant correlations between the ASR amplitudes and PPI levels in the low-responder group, and a significant negative correlation was identified in the intermediate- and high-responder groups. Further analysis of group comparisons indicated that the lowest responder group (Group 1) showed the lowest levels of PPI at the 110 dB startle stimulus compared with the other responder groups (Groups 2–8), and at the 120 dB startle stimulus, the higher responder groups (Groups 7′ and 8′) exhibited lower levels of PPI than the other groups (Groups 1′-6′), as expected from the results of the correlation analysis. Collectively, these results indicate that basal startle reactivity is associated with PPI levels in C57BL/6J mice.

The preceding study by Yee et al. [23], in which a cohort of 102 C57BL/6J mice was subdivided into three groups on the basis of ASR amplitudes, showed that there were significant positive correlations between ASR amplitudes and PPI levels in the groups of mice that exhibited lower and intermediate ASR levels at 100-dB and 110-dB stimuli, whereas no significant correlation was identified in the group of mice with higher ASR levels. Their findings of a positive correlation between the two measures in the lower responders to the smaller acoustic startle stimulus appear to be consistent with our results. Yee et al. [23] also reported that at a 120-dB stimulus, there was a significant positive correlation between ASR amplitudes and PPI levels in the low-responder group, whereas a negative but non-significant correlation between the two measures was identified in the intermediate-responder groups; no correlation was identified in the high-responder group. Similar results were obtained in our study, in which small positive but non-significant correlations between the amplitude of the ASR to the 120-dB stimulus and PPI levels were identified in the lower responders, and significant negative correlations between the two measures were identified in the intermediate or higher responders to 120-dB. Overall, our meta-analytic study of a large number of C57BL/6J mice, in which the correlation patterns identified are generally similar to the patterns obtained in the previous findings by Yee et al. [23], strengthens the reliability of the findings regarding the relationships between basal startle reactivity and percentages of PPI in C57BL/6J mice.

**Fig. 4:**
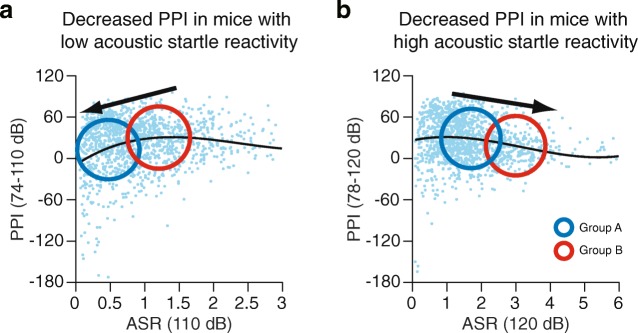
Problematic cases of PPI results that may be confounded by basal startle reactivity. Two representative cases of PPI results in two groups of mice with different levels of basal startle reactivity are shown for 74–110 dB (**a**) and 78–120 dB (**b**) stimuli. Lines indicate regression curves of the acoustic startle response and PPI for each trial type. Blue and red circles indicate clusters of PPI data in different groups of mice with lower or higher levels of ASR (Groups A and B). The two cases may impose limitations on the interpretation of PPI results: as expected from the results of the correlation analysis of ASR and PPI, differences in PPI levels between two groups of mice may result from a low startle reactivity (**a**) or a high startle reactivity (**b**) in either group

Our study indicates that basal startle reactivity of animals requires careful attention in interpreting differences in PPI between groups of mice, or experimental and control groups, because basal startle reactivity may be one confounding factor that affects PPI levels. As illustrated by examples of potential outcomes of PPI tests in two groups of mice with different levels of basal startle reactivity in Fig. 4, there are cases in which the PPI results from the two groups may be confounded by their basal startle reactivity. For example, if mice in one group show a low startle response to a low intensity acoustic startle stimulus, their PPI levels may be lower than the levels of mice that show a higher startle response (Fig. 4a), according to our results of the correlation analyses and group comparisons (Figs. 1, 2 and 3). In general, the ASR and its PPI decrease with increasing age, which is associated with declines in hearing ability and motor function [16, 17, 26]. The lower PPI levels in the group of the low startle responders may be a result of their low reactivity to sound stimuli or potentially low hearing and/or motor function. In a low responder group, their basal startle responses are not expected to be further suppressed by the prepulse stimuli, which may lead to low PPI levels. Thus, the PPI experiment in animals showing low hearing and/or motor function, such as aged animals (Additional file 3: Figure S2), is potentially limited in assessing their sensorimotor gating functions, and it would be desirable to use young mice in these experiments. In another case (Fig. 4b), mice that show a high ASR may exhibit lower PPI levels than mice with a lower ASR due to a ceiling effect in that the intensity of a weak prepulse stimulus may be insufficient to suppress their high startle responses because of their high reactivity to the auditory stimulus. There is also a possibility that no differences in PPI levels between groups may occur from both the low and high startle reactivity of the groups. In these cases, PPI results may depend on hearing and/or motor function in the mice of the groups and may not directly reflect sensorimotor gating function. These findings on representative examples seem to highlight the possible effects of different intensities in the startle stimulus on PPI results. Thus, researchers should consider evaluating ASR and PPI using different intensities of startle stimuli in order to identify potential phenotypes in ASR and PPI. It is important to note that the present study showed that there were no significant differences in PPI levels among groups of mice that showed startle responses within a certain range of amplitudes (Fig. 3). Thus, PPI results may be simply explained by differences in sensorimotor gating function when comparing PPI levels between groups that show ASRs within a specific range of ASR amplitudes. In our laboratory, we have assessed a wide range of behaviors through a battery of behavioral tests in a substantial number of strains of genetically engineered mice, and substantial behavioral data, which have previously been published, are available in our online database (Mouse Phenotype Database, http://www.mouse-phenotype.org). Using the data of ASR and PPI in mutant strains of mice with a C57BL/6J background from the database [27–29], an examination of the scatter plots of the two measures suggests that in some cases of mutant strains, their startle reactivity may need to be considered as a confounding factor that affects PPI results (Additional file 4: Figure S3; one example is that *Gria4*^−/−^ mice with a low ASR amplitude showed low PPI levels, refer to Sagata et al. [27] for a discussion). These observations indicate that a detailed examination of scatter plots of the ASR and PPI may be useful for the assessment of PPI characteristics in each group and the interpretation of PPI results.

Variations in PPI may be caused by interactions of multiple genetic and environmental factors, and the precise causes of the variations remain unclear. In this study, the large-scale meta-analysis of data from more than 1300 C57BL/6J male mice indicates that basal acoustic startle reactivity, which is associated with variations in PPI levels, may be a confounding factor for PPI results. Furthermore, additional correlation analyses of ASR latency and PPI revealed that mice showing a longer ASR latency tend to exhibit lower ASR amplitudes and lower PPI levels, indicating that differences in latency to ASR can also be a potential confounding factor in evaluating PPI. The analyses shown in scatter plots of basal ASR measures and PPI levels may facilitate an understanding of the relationships between the two behavioral measures in mice. Thus, our study indicates the importance of describing the details of behavioral characteristics in each group examined in this test paradigm to draw a convincing conclusion when interpreting PPI results.

## Additional files

Additional file 1: Statistical results of group comparisons of percent PPI in C57BL/6J mice. (XLSX 167 kb)

Additional file 2: **Figure S1.** Scatter plots of the latency to peak of acoustic startle response and percentages of prepulse inhibition of the startle response in male C57BL/6J mice. Relationships between behavioral measures were assessed by Spearman’s rank correlation coefficients (Rho) and *p* values in 721 mice (2–3-month old, *n* = 423; 4–5-month old, *n* = 198; 6–7-month old, *n* = 64; 8–12-month old, *n* = 36). Scatter plots of the latency to peak of startle response and amplitude of startle response at 110-dB (A) and 120-dB (D) stimuli are shown, and scatter plots of the latency to peak of startle response and percentage of prepulse inhibition at 74–110 dB (B), 78–110 dB (C), 74–120 dB (E), and 78–120 dB (F) trials are presented. (PDF 530 kb)

Additional file 3: **Figure S2.** Scatter plots of the amplitudes of acoustic startle response and percentages of prepulse inhibition of the startle response in different ages of male C57BL/6J mice. Relationships between acoustic startle responses to 110- and 120-dB pulse stimuli and percentages of prepulse inhibition were assessed by by Spearman’s rank correlation coefficients (Rho) and p values in 1363 mice in total (2–3-month old, *n* = 757; 4–5-month old, *n* = 389; 6–7-month old, *n* = 167; 8–12-month old, *n* = 50). Scatter plot of the amplitudes of startle response to pulse stimulus and percentages of prepulse inhibition in each age groups of mice at 74–110 dB (A–E), 78–110 dB (F–J), 74–120 dB (K–O), and 78–120 dB (P–T) trials. (PDF 1237 kb)

Additional file 4: **Figure S3.** Examples of relationships between the acoustic startle response and prepulse inhibition in mutant and wild-type mice. (A–C) Scatter plots of acoustic startle amplitudes and percentages of prepulse inhibition in mutant strains of mice with a C57BL/6 background (for *Apc*^1638T/1638T^ mice, Onouchi et al., 2014; for *Gria4*^−/−^ mice, Sagata et al., 2010; for *Zfhx2*^−/−^ mice, Komine et al., 2012) that were obtained from the Mouse Phenotype Database (http://www.mouse-phenotype.org) show that the interpretation of differences in PPI levels between mutant and wild-type mice may be confounded by differences in basal startle reactivity (refer to Discussion and Fig. 4). The scatter plots suggest that differences in PPI levels between *Apc*^1638T/1638T^ and *Apc*^+/+^ mice and between *Gria4*^−/−^ and *Gria4*^+/+^ mice may result from low startle reactivity in mutants (A, B), and the lower levels of PPI in *Zfhx2*^−/−^ may be a result of their higher startle reactivity (C). (PDF 472 kb)

## Abbreviations

ASR: Acoustic startle response
PPI: Prepulse inhibition
