# Supplementary figures and images for: Relationships between the acoustic startle response and prepulse inhibition in C57BL/6J mice: a large-scale meta-analytic study
Source: Mol Brain. 2018 Jul 13;11:42. doi: 10.1186/s13041-018-0382-7 (PMC6044095; doi:10.1186/s13041-018-0382-7)

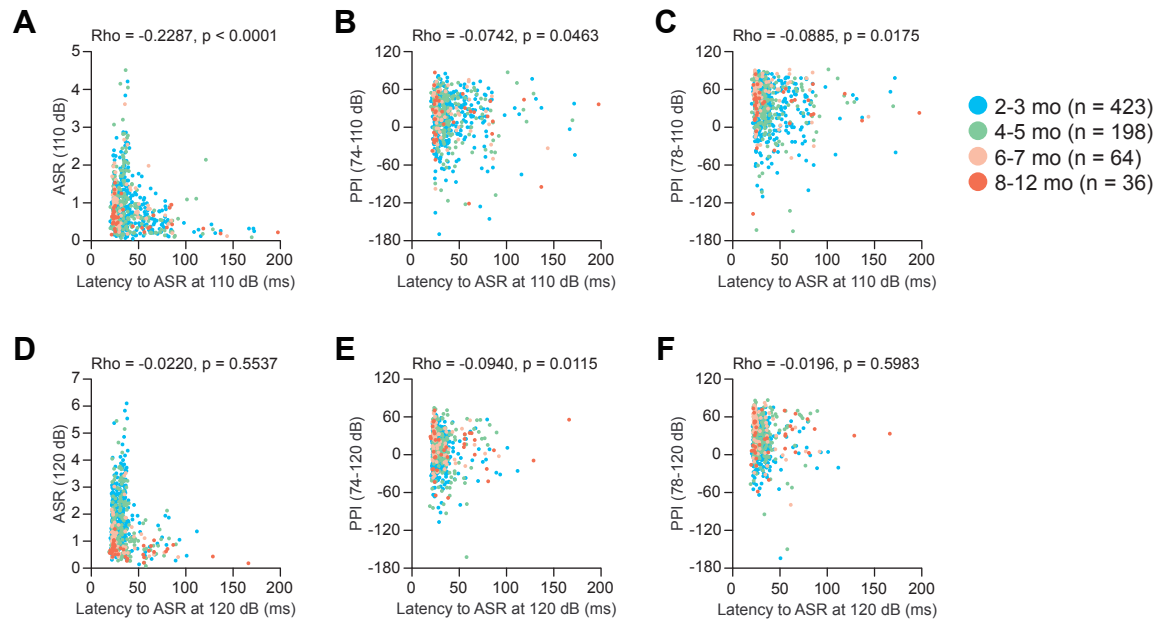

Supplement: Supplementary file 2 — Figure S1. Scatter plots of the latency to peak of acoustic startle response and percentages of prepulse inhibition of the startle response in male C57BL/6J mice. Relationships between behavioral measures were assessed by Spearman’s rank correlation coefficients (Rho) and p values in 721 mice (2–3-month old, n = 423; 4–5-month old, n = 198; 6–7-month old, n = 64; 8–12-month old, n = 36). Scatter plots of the latency to peak of startle response and amplitude of startle response at 110-dB (A) and 120-dB (D) stimuli are shown, and scatter plots of the latency to peak of startle response and percentage of prepulse inhibition at 74–110 dB (B), 78–110 dB (C), 74–120 dB (E), and 78–120 dB (F) trials are presented. (PDF 530 kb) [file 13041_2018_382_MOESM2_ESM.pdf]

● 2-3 mo (n = 757)  
● 4-5 mo (n = 389)  
● 6-7 mo (n = 167)  
● 8-12 mo (n = 50)

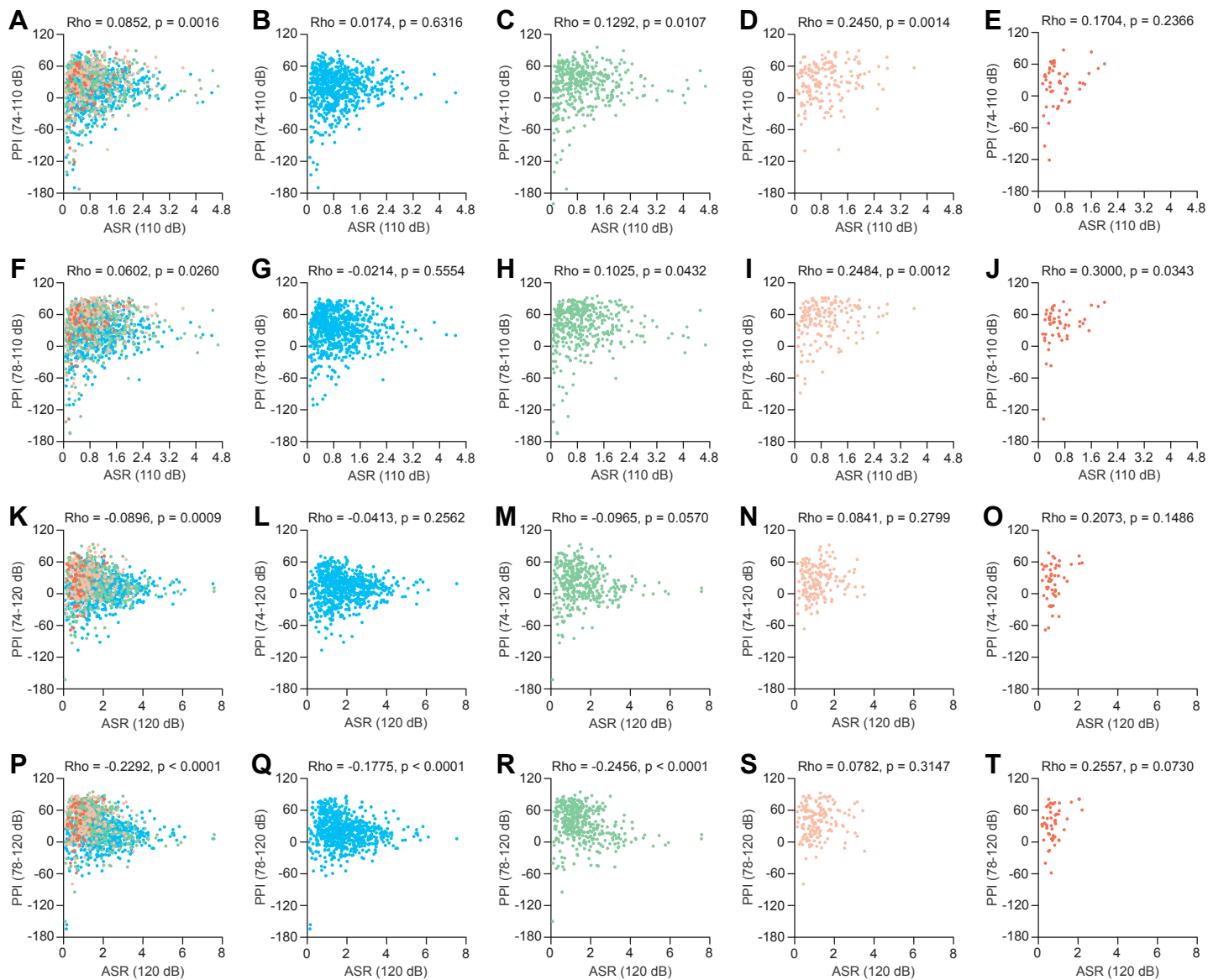

Supplement: Supplementary file 3 — Figure S2. Scatter plots of the amplitudes of acoustic startle response and percentages of prepulse inhibition of the startle response in different ages of male C57BL/6J mice. Relationships between acoustic startle responses to 110- and 120-dB pulse stimuli and percentages of prepulse inhibition were assessed by by Spearman’s rank correlation coefficients (Rho) and p values in 1363 mice in total (2–3-month old, n = 757; 4–5-month old, n = 389; 6–7-month old, n = 167; 8–12-month old, n = 50). Scatter plot of the amplitudes of startle response to pulse stimulus and percentages of prepulse inhibition in each age groups of mice at 74–110 dB (A–E), 78–110 dB (F–J), 74–120 dB (K–O), and 78–120 dB (P–T) trials. (PDF 1237 kb) [file 13041_2018_382_MOESM3_ESM.pdf]

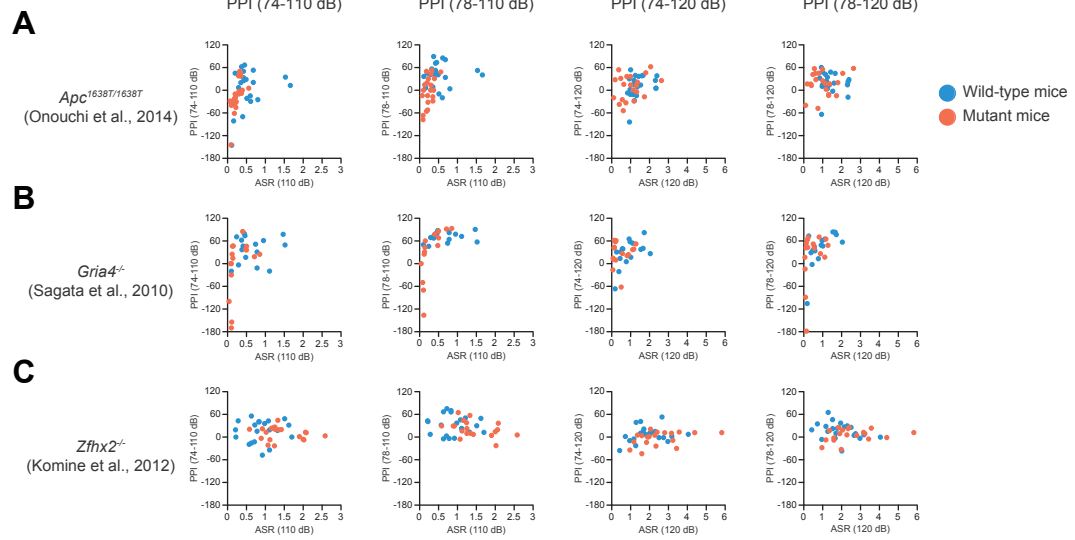

Supplement: Supplementary file 4 — Figure S3. Examples of relationships between the acoustic startle response and prepulse inhibition in mutant and wild-type mice. (A–C) Scatter plots of acoustic startle amplitudes and percentages of prepulse inhibition in mutant strains of mice with a C57BL/6 background (for Apc1638T/1638T mice, Onouchi et al., 2014; for Gria4−/− mice, Sagata et al., 2010; for Zfhx2−/− mice, Komine et al., 2012) that were obtained from the Mouse Phenotype Database (http://www.mouse-phenotype.org) show that the interpretation of differences in PPI levels between mutant and wild-type mice may be confounded by differences in basal startle reactivity (refer to Discussion and Fig. 4). The scatter plots suggest that differences in PPI levels between Apc1638T/1638T and Apc+/+ mice and between Gria4−/− and Gria4+/+ mice may result from low startle reactivity in mutants (A, B), and the lower levels of PPI in Zfhx2−/− may be a result of their higher startle reactivity (C). (PDF 472 kb) [file 13041_2018_382_MOESM4_ESM.pdf]
